# Is This Inflammation, Conjunctiva-Associated Lymphoid Tissue, or Tertiary Lymphoid Structures in These Rabbits?

**DOI:** 10.1167/tvst.13.12.3

**Published:** 2024-12-03

**Authors:** JoAnn C. L. Schuh

**Affiliations:** 1 Retired consultant (toxicologic pathology), Bainbridge Island, WA, USA. e-mail: joanncl.schuh@gmail.com

The induced disease rabbit model of pterygium reported by Rodriguez-Barrientos et al.^1^ in “Key Clinical and Histopathological Features of a Pterygium-Like Induced Lesion in a Rabbit Model,” recently published in *Translational Vision Science & Technology*, could be a valuable nonclinical surgical or therapeutic interventional model, but the histopathology findings are indicative of conjunctiva-associated lymphoid tissue (CALT) rather than inflammation.

The authors induced pterygium-like lesions by unilateral subconjunctival injection of fibroblasts and Matrigel into the bulbar conjunctiva near the limbus of New Zealand White rabbits, with histopathology restricted to day 23. The resulting clinically progressive pterygium-like lesions in 60% of the eyes consisted of hyperemia and enlarged vasculature that began to diminish by day 15, as well as conjunctival changes of an elevated lesion that began at days 7 to 10, became flat by day 18, and appeared as a white–yellow elevated lesion on day 23. The histopathology analysis was presented as representative photomicrographs of graded lesions in their Figure 3, but the lack of focus and white balance issues, apparent loss of the conjunctival epithelial surface in some sections, and lack of a higher magnification photomicrograph to confirm the inflammatory nature of the cell infiltrates are problematic. Regardless, I was immediately struck that the description and photomicrographs were consistent with post-injection induction of CALT rather than active inflammation. Confusingly, the authors use both inflammation and cell infiltrate to describe the conjunctival response. These morphological descriptors are not interchangeable, as cell infiltrates consist predominantly of one or sometimes multiple cell types, but in the absence of degenerative or vascular changes that typify tissue inflammation^2^. All but the photomicrographs in their Figures 3A and 3B show small (Figs. 3C, 3E) to large (Figs. 3D, 3F–3I) accumulations of apparently mononuclear cells in the submucosal region of the conjunctiva, with some cells being widely scattered (Fig. 3C) and others forming dense accumulations (Figs. 3D–3I), which are organizing (Figs. 3G–3I) into a structure consistent with lymphoid follicles (Figs. 3H, 3I, and maybe Figs. 3F and 3G), including pale germinal centers (Figs. 3H, 3I). The authors also describe grade 4 lesions organizing as multifocal (Fig. 3G), lymphoid mass (Fig. 3H), and granuloma (Fig. 3I). In Figure 3I, there is no histopathology evidence of a granuloma, but the appearance is consistent with a lymphoid follicle with a germinal center, so this morphological diagnosis must be reconsidered. Overall, the authors’ documentation of grossly raised lesions that correlate microscopically to one or multiple organized lymphocytic masses is more consistent with CALT than inflammation. This is further supported by the histological reduction in epithelial height and reduced numbers of goblet cells over the lymphoid masses (Figs. 3F, 3G, upper right; Fig. 3H). These epithelial changes are consistent with the flattened and specialized microfold cells with weak intercellular junctions that overlie mucosa-associated lymphoid tissue (MALT) to allow intraepithelial leukocytes to sample antigens and promote local immune response at mucosal surfaces. Another less likely diagnostic consideration in this study would be formation of tertiary lymphoid structures (TLSs), which are lymphoid follicles induced in nonlymphoid tissues, usually concurrent with chronic inflammation.^2^^,^^3^

CALT is the most well-described MALT around the eyes of humans and large laboratory animals. These CALTs can be loose accumulations of lymphocytes or multifocal dense masses organized into discrete lymphoid follicle structures, with or without pale central germinal centers.^3^ In normal rabbits, CALT has been identified as early as 11 days postnatally.^4^^,^^5^ Although CALTs are most frequently found in the palpebral conjunctiva (and nictitating membrane in animals) and less frequently near the fornix and bulbar conjunctiva in most species, a variety of microorganisms, corneal transplantations, pharmaceuticals, and topical ocular stimuli can induce and expand CALT formation in all conjunctival locations.^3^ In the absence of confirmed inflammatory changes, particularly after antigenic stimuli, organized lymphoid accumulations as described in this paper should be reported as CALT rather than as inflammation or TLSs.^3^ To assume that lymphocyte infiltrates in tissues are evidence of previous inflammation with restoration of normal tissue architecture is also speculative and a difficult diagnosis to support, particularly in mucosal sites that form MALT.^3^

In order to justify this induced rabbit model of pterygium-like lesions, the authors need to provide additional histopathology characterization for the cellular and tissue responses, including differentiating inflammation from infiltrates, CALT, and TLSs. Use of research animals as a surrogate for interventional therapy or surgery of human disease requires great care to optimize study design and provide detailed characterization of histopathology prior to making evidence-based conclusions. In the reported study, this would have been best accomplished by a time course study to document conjunctival changes at multiple time points, such as on days 3, 7, 15, 23, and 30. I would also recommend consulting with a veterinary pathologist specializing in eyes to provide support on study design, optimizing tissue preparation, and histopathology terminology and interpretation in animals. Sectioning of the entire eye with attached conjunctiva (en bloc) would have provided optimal histopathology examination of the conjunctival response^3^ in this study.
